# C- and O-Band Dual-Polarization Fiber-to-Chip Grating Couplers for Silicon Nitride Photonics

**DOI:** 10.1021/acsphotonics.3c00834

**Published:** 2023-08-30

**Authors:** Manuel Kohli, Daniel Chelladurai, Boris Vukovic, David Moor, Dominik Bisang, Killian Keller, Andreas Messner, Tatiana Buriakova, Michael Zervas, Yuriy Fedoryshyn, Ueli Koch, Juerg Leuthold

**Affiliations:** † ETH Zurich, Institute of Electromagnetic Fields (IEF), 8092 Zürich, Switzerland; ‡ Ligentec SA, 1024 Ecublens, Switzerland

**Keywords:** integrated photonics, fiber-to-chip coupling, grating, silicon nitride

## Abstract

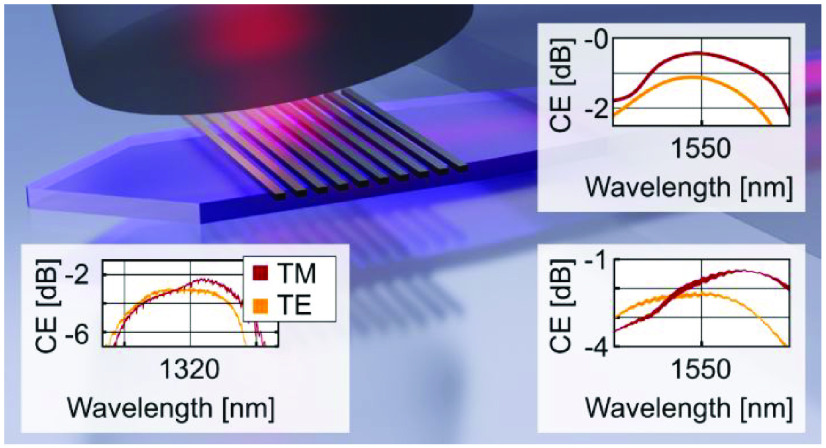

Highly efficient coupling of light from an optical fiber to silicon nitride (SiN) photonic integrated circuits (PICs) is experimentally demonstrated with simple and fabrication-tolerant grating couplers (GC). Fully etched amorphous silicon gratings are formed on top of foundry-produced SiN PICs in a back-end-of-the-line (BEOL) process, which is compatible with 248 nm deep UV lithography. Metallic back reflectors are introduced to enhance the coupling efficiency (CE) from −1.11 to −0.44 dB in simulation and from −2.2 to −1.4 dB in experiments for the TE polarization in the C-band. Furthermore, these gratings can be optimized to couple both TE and TM polarizations with a CE below −3 dB and polarization-dependent losses under 1 dB over a wavelength range of 40 nm in the O-band. This elegant approach offers a simple solution for the realization of compact and, at the same time, highly efficient coupling schemes in SiN PICs.

## Introduction

Fiber-to-chip coupling should offer maximum efficiency for high-performance applications while being simple and fabrication tolerant to achieve scalability and allow rapid prototyping. Silicon nitride photonics has gained attention in the past decades due to numerous advantages, such as propagation losses below 0.1 dB/m, dispersion engineering, negligible two-photon absorption, wide transparency window, low thermal sensitivity, and low sensitivity to fabrication variations.^1−7^ Yet, a simple and efficient fiber-to-chip coupling scheme compatible with the SiN process flow is still missing.

To couple light efficiently to the SiN platform, two state-of-the-art methods exist: edge coupling (EC) and grating coupling (GC). EC, on the one hand, is typically known for its high performance in terms of CE, bandwidth, and polarization insensitivity.^8^ EC on SiN further benefits from the low index contrast of the waveguides, making it easier to match the modal size between on-chip waveguides and the optical fiber, with CEs reaching below 1 dB.^9−11^ GC, on the other hand, allows out-of-plane coupling and therefore wafer-level testing, eliminating the need for intensive processing of a sensitive optical facet at the edges of the chips while typically exhibiting relaxed fiber alignment tolerances.^8^ The main drawbacks of GC compared to EC, however, are the lower bandwidth, lower coupling efficiency, and high polarization dependency. SiN grating couplers have an improved bandwidth in comparison to silicon ones due to the lower index contrast,^12^ allowing 1 dB bandwidths of up to 80 nm.^13^ The second challenge of low CE, however, is further hampered on SiN, as lower index contrast results in a lower scattering strength, yielding CEs of −4.2 dB in single-etch SiN gratings.^12^ To address this, different approaches have been proposed and demonstrated. Most commonly, multilayer distributed Bragg reflectors^14−17^ consisting of silicon layers beneath the SiN waveguide are employed, yielding CEs as low as −1.17 dB in measurement and −0.31 dB in simulation with a 40 nm 1 dB bandwidth.^14^ A different approach consists of defining grating bars on multiple layers,^13,18−21^ requiring precise planarization and lithographical alignment. Experimental results show CE of −1.29 dB and a high 1 dB bandwidth of 80 nm^13^ and simulations of −0.39 dB.^21^ A simpler dual-layer GC is demonstrated with a SiN grown on SOI yielding −2.5 dB CE in the C-band,^19^ although deep trenches through a triple-layer consisting of SiN, SiO_2_, and Si must be etched. Utilizing the self-imaging method, −1.5 dB CE has been achieved with a staircase grating and a fiber placement over 100 μm above the surface.^22^ Overcoming the third challenge of polarization dependency is vital for applications, such as on–off keying receivers in optical communications, where polarization of the incoming light is not known a priori.^23^ Solutions are more difficult, however, as matching of the Bragg condition for two polarizations at the same time is hindered by the typical birefringence of planar waveguides. Possible solutions include 2D grating couplers demonstrated on silicon,^24,25^ that typically split the polarizations into two different directions. For 1D grating couplers, only a few examples exist, such as combining two grating periods with an experimental CE of −7.8 dB and a polarization dependent loss (PDL) of 0.8 dB over 20 nm^26^ or a multilayer grating consisting of two SiN and one silicon layer with a demonstrated CE of −4.8 dB and 1dB PDL of over 100 nm.^18^ Despite the impressive grating coupler demonstrations, a method that includes fabrication tolerance and simplicity suited for rapid prototyping, while also achieving low loss in the C- and O-bands and polarization insensitivity, is still missing on SiN.

**Figure 1:**
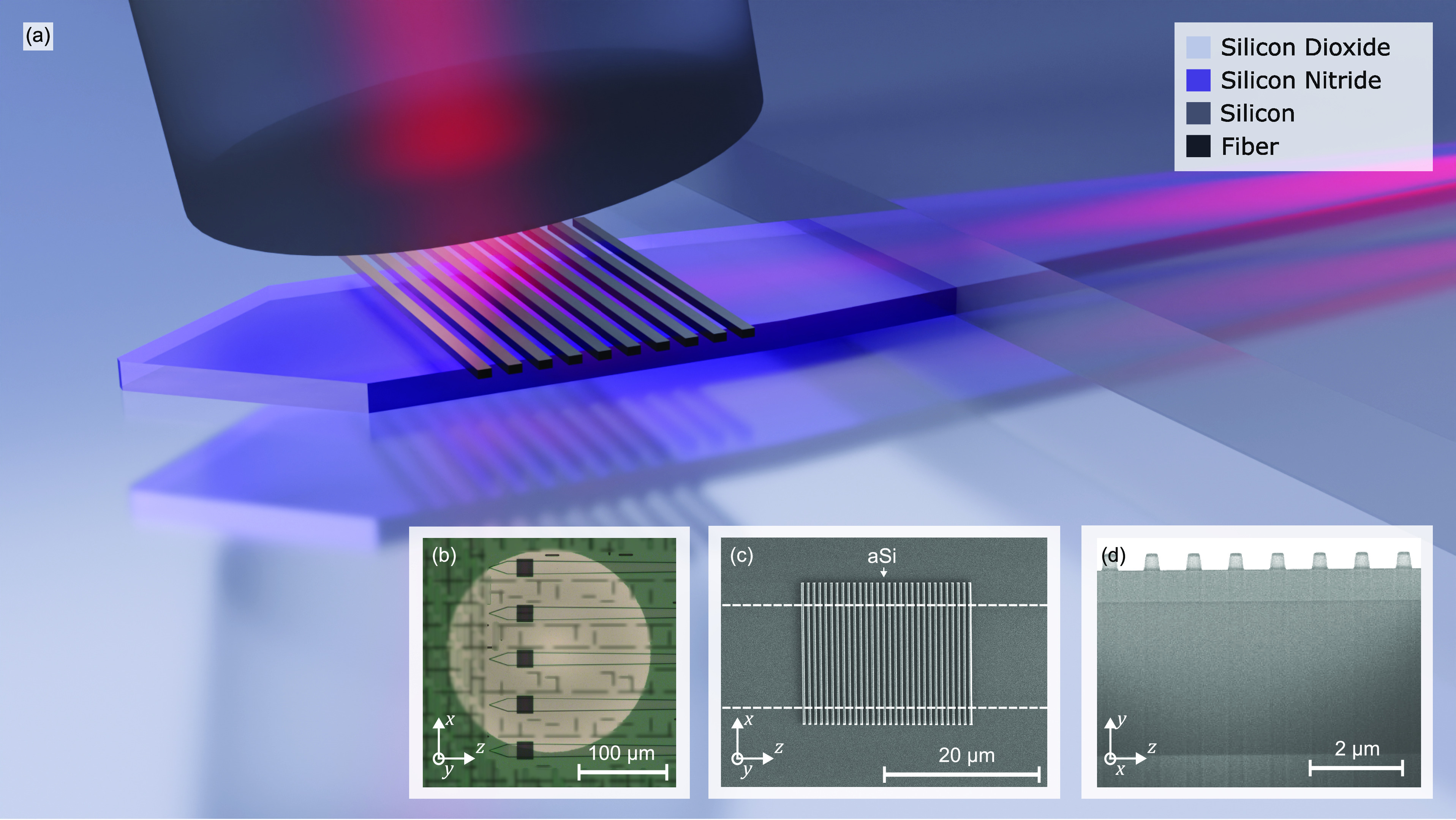
Fabrication-tolerant back-end-of-the-line amorphous silicon overlay grating couplers for the SiN platform. (a) Three-dimensional illustration of the a-Si overlay grating couplers with back reflector. (b) Optical microscope image of fabricated couplers with a circular back reflector. (c) SEM image of couplers without a backside mirror. (d) SEM image of a FIB-generated cross section of the C-band grating coupler without back reflector.

In this work, we introduce a simple and fabrication tolerant method to couple light efficiently into foundry-produced SiN waveguides with silicon overlay gratings; see Figure 1. Coupling efficiencies in the C-band as high as −2.2 dB in measurement and −1.11 dB in simulation are demonstrated, and we show improvement to −1.4 dB in measurement and −0.44 dB in simulation by depositing a metal back reflector without changing any other parameter. The versatility of the approach is further demonstrated with a polarization insensitive design in the O-band reaching −2.2 and −3 dB CE for TM and TE polarizations, respectively. The PDL remains below 1 dB over 40 nm.

The work in this paper is in part based on our results first published at the Optica APC congress in 2021 and 2022.^27,28^

## Concept

**Figure 2:**
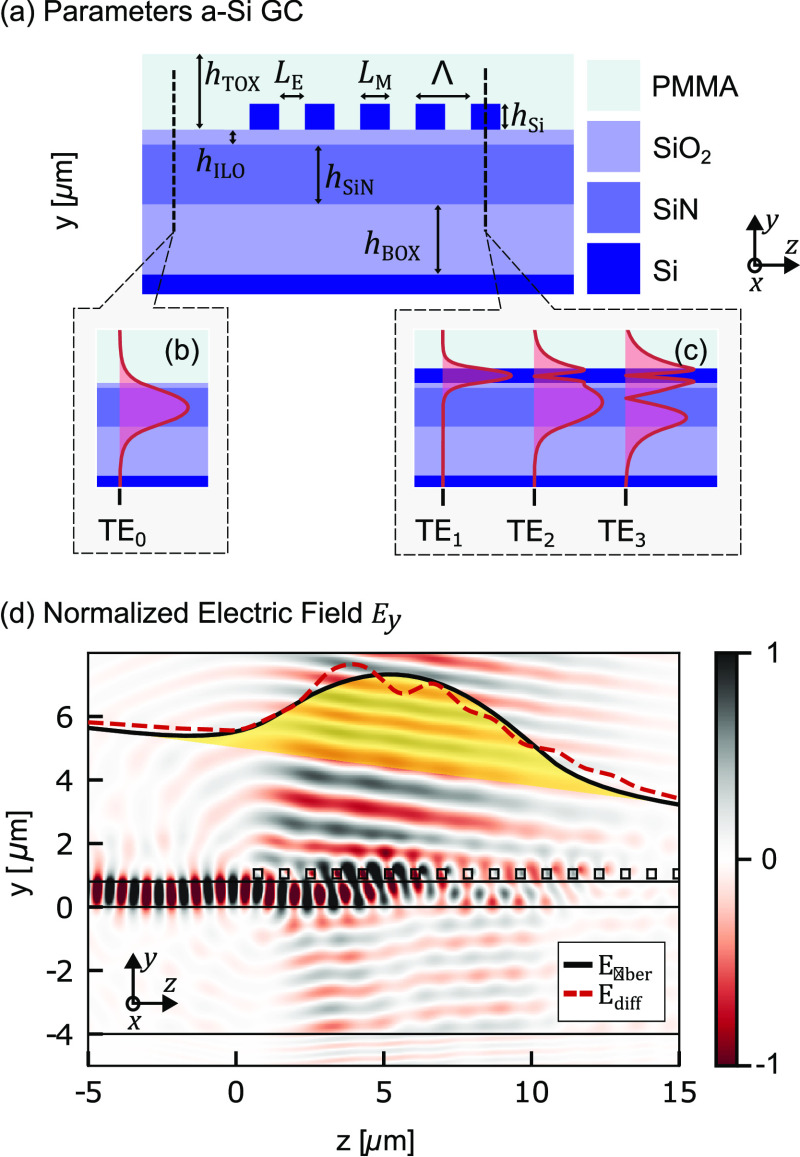
(a) Schematic of the grating coupler with the relevant parameters. (b) Mode in the SiN waveguide without overlay. (c) Modes in the multilayer structure, which have an influence on the diffraction. (d) Normalized electric field in *y*-direction of the suggested grating coupler, showing that there is an interaction between the different modes.

The grating coupler presented in this work consists of amorphous silicon (a-Si) grating bars on top of an 800 nm thick SiN layer separated by a thin interlayer oxide (ILO) between the a-Si and the SiN, see Figure 2a. The overlay approach differs from the usual concept, where the grating is within the SiN waveguide. In this approach, we exploit the Bragg diffraction coupling by placing a grating in the a-Si overlay layer. Yet, we also exploit the multimode section in the grating; see Figure 2c. The multimode section enables a diffraction in a Gaussian-like mode profile along the grating section.

Conceptionally, one can understand the coupling scheme from Figure 2d. First, a fundamental TE_0_ mode is propagated from left to right in the SiN waveguide. A sketch of the mode profile is given in Figure 2b. As it is propagating into the multimode grating section, the mode is mapped onto the three eigenmodes (TE_1_, TE_2_, TE_3_) of the multimode section; see Figure 2c. Initially, the center of the gravity of the light is in the SiN waveguide layer at the bottom. Only a small fraction of the mode is in the overlay layer and is diffracted. As the three modes propagate in the grating multimode section, the modes constructively interfere into the overlay layer, and accordingly, there is stronger diffraction into free space. At some point, the mode fades away and the diffraction decreases. Simulations confirm that this gradual increase and decrease of emission results in an almost ideal excitation of a Gaussian mode profile; see Figure 2d, which is close to the Gaussian mode profile of the fundamental mode in a single mode fiber.

In the next step, we can derive an approximate value for the pitch Λ of the grating. Toward this end, we follow the general design principles of a 1D grating coupler. To diffract the incoming light into a fiber, the phase matching condition or Bragg condition, which relates the wave vectors of the incident beam and the diffracted beams, must be fulfilled. This can be expressed as
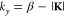
1where *k*_*y*_ = 2π·sin(θ)·*n*_cladding_/λ is the wave vector component in *y*-direction, i.e., the out-of-plane wave, β = 2π·*n*_GC_/λ is the propagation constant of the multimode waveguide and |**K**| = 2π/Λ the grating vector. Assuming a diffraction angle θ toward the normal axis, the phase-matching equation can be rewritten to
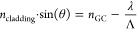
2where *n*_GC_ is the effective index of the waveguide grating section, *n*_cladding_ is the refractive index of the cladding, θ is the diffraction angle, λ the center wavelength, and Λ the pitch of the grating.^8^

This equation can be solved for Λ if the effective index of the multimode grating section were known. Here, we approximate *n*_GC_ by averaging the effective index of the etched and unetched region weighted by the fill factor (FF):
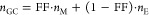
3where *n*_M_ is the effective index in the unetched region, *n*_E_ is the effective index in the etched region, and FF = *L*_M_/(*L*_M_ + *L*_E_) is the fill factor. To apply this approximation to a multimode structure, see Figure 2c, the effective index *n*_M_ of the unetched part in the grating waveguide can be approximated by a weighted average of the effective indices of the respective modes.^18^ The relative weights are given as the fractions by which each mode is excited.

**Figure 3:**
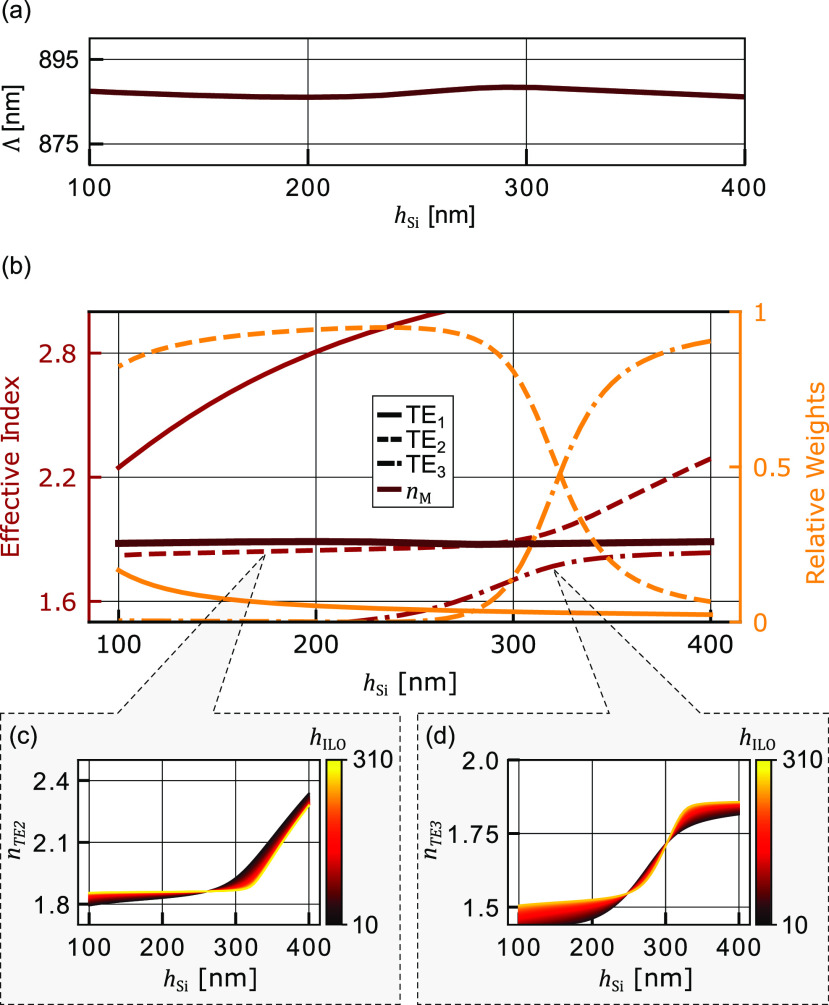
(a) Λ calculated using the weighted average, showing a near constant value in relation to *h*_Si_. (b) *n*_eff_ (left) and the relative weights to TE_0_ (right) of the modes TE_1_, TE_2_, and TE_3_ in relation to *h*_Si_. The weighted approximation of *n*_M_ is near constant, indicating a large tolerance on to *h*_Si_. Simulated to *n*_TE2_ (c) and *n*_TE3_ (d) in relation to *h*_Si_ with the colors indicating *h*_ILO_. *h*_ILO_ has a minimal influence on the effective index.

Figure 3a shows the calculated Λ in relation to *h*_Si_ using the weighted average method with an FF of 0.32. We find a nearly constant value despite a large variation in *h*_Si_. This shows a large tolerance of the grating coupler toward a variation of *h*_Si_. Following, we investigate the origin of this fabrication tolerance. Toward this goal, we plot in Figure 3b, the effective index (left) and the relative weights (right) of modes TE_1_, TE_2_, and TE_3_ from Figure 2c. The relative weights is calculated using eigenmode expansion and refers to the overlap between the three modes to TE_0_, see Figure 2b. Although the individual modes change considerably with *h*_Si_, we find that the effective index of the unetched region *n*_M_ to be near constant for a large range of *h*_Si_ values. This is an interesting finding as *n*_M_ is the weighted average of the modes. Next we investigate the variation of *n*_M_ with the interlayer-oxide thickness (*h*_ILO_). Figure 3c shows the effective index of TE_2_ and (d) of TE_3_ in relation to *h*_Si_. The colors indicate *h*_ILO_. It can be seen that *h*_ILO_ has little influence on the effective index of the two modes. This leads to the conclusion that this a-Si overlay grating coupler has a high tolerance on both *h*_Si_ and *h*_ILO_.

## Experimental and Methods

### Fabrication Tolerant C-Band Grating Coupler

In this section, we show the simulation results of the C-band grating coupler for a maximum CE of −0.42 dB. We analyze the fabrication parameters to show the large fabrication tolerance of our approach. The phase-matching condition allows us to pick a reasonable starting condition for the grating around which we can apply a thorough 2D FDTD analysis. The optimization routine has led us to an optimized grating coupler with parameter values of *h*_Si_ = 290 nm, *h*_ILO_ = 100 nm with a pitch of Λ = 888 nm and fill factor of FF = 0.32. The thicknesses of the SiN (*h*_SiN_ = 800 nm) and buried oxide (*h*_BOX_ = 4.1 μm) are kept constant during the optimization.

**Figure 4:**
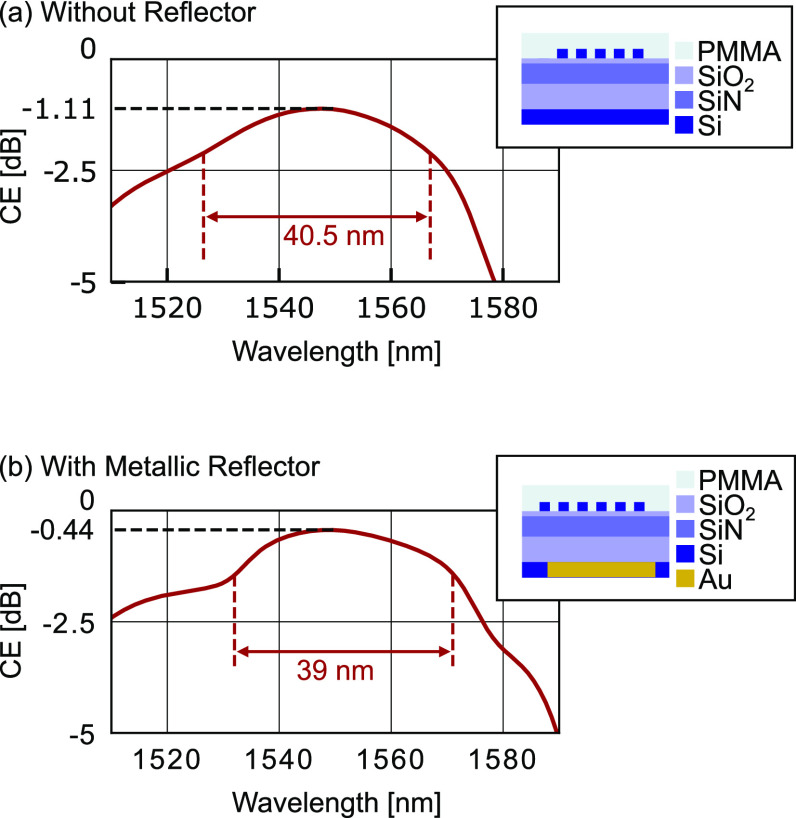
Simulated coupling efficiency for (a) a-Si overlay grating and (b) a-Si overlay grating with metallic mirror. The directionality can be improved by including a metallic mirror, increasing the coupling efficiency from −1.11 to −0.44 dB.

The wavelength tolerance of the coupling efficiencies of the SiN waveguide toward a SMF fiber tilted by an angle of 7° is shown in Figure 4. The coupling efficiency of a grating coupler without a back reflector is plotted in Figure 4a. The simulated grating is formed only in the a-Si overlay layer and shows a CE of −1.11 dB with a 1 dB bandwidth of 40.5 nm. Simulations show that within 20 grating bars almost 100% of the light is diffracted. For the upward diffracted field there is a 96.6% (−0.15 dB) modal overlap with the fiber. This leaves the reduction of the downward diffracted light as a main method of improvement.

Indeed, it is found that the CE can be further increased to −0.44 dB by adding a metallic back reflector below the buried oxide (4.1 μm below the SiN); see Figure 4b. There is no need for changing any parameters of the grating coupler. The 1 dB bandwidth stays large with 39 nm.

**Figure 5:**
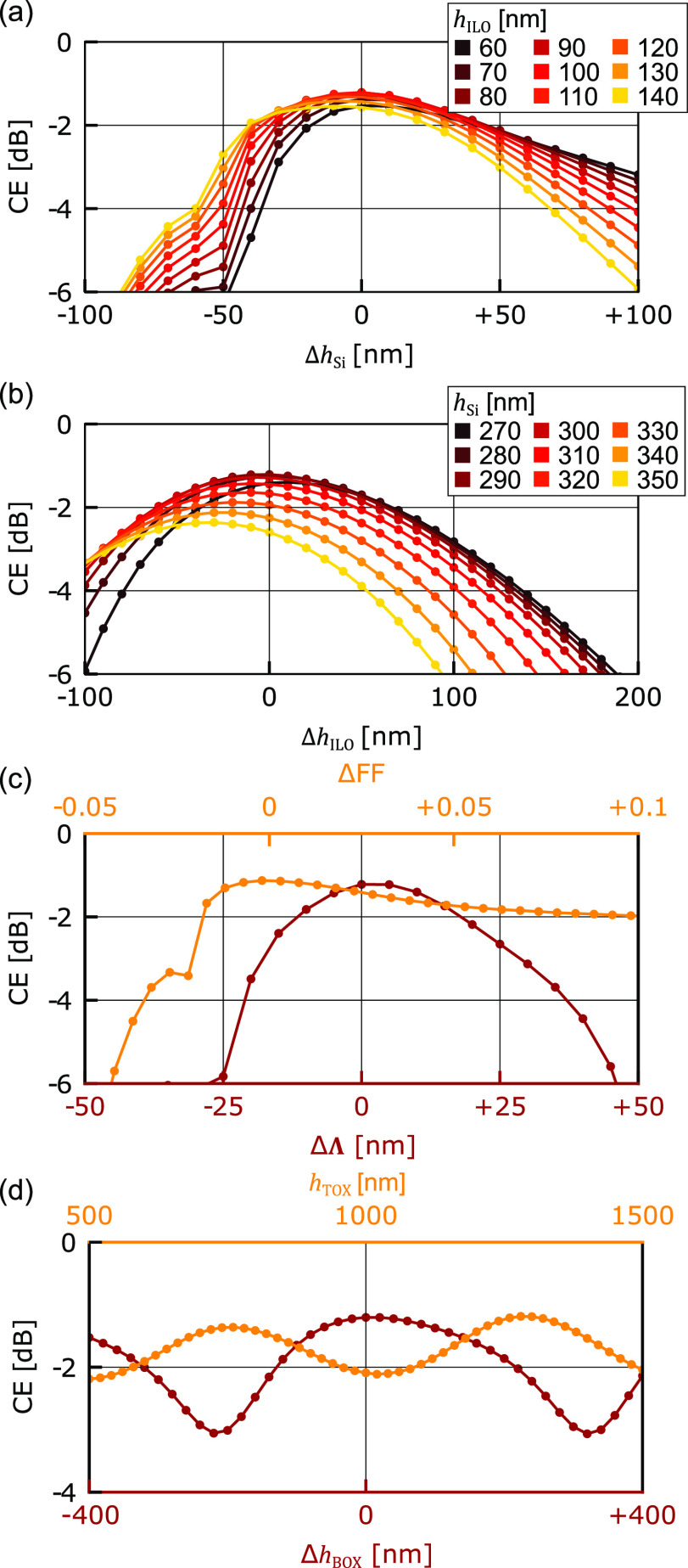
Simulations showing that the suggested grating coupler offers large fabrication tolerance. (a, b) Simulated CE with varying *h*_Si_ and *h*_ILO_ showing large fabrication tolerance with a 1 dB tolerance of 86 and 139 nm, respectively. (c) Tolerance sweeps over Λ (red line) and FF (yellow line) with a 1 dB tolerance of 34 nm for the Λ and 0.1455 for the FF. (d) CE in relation to *h*_BOX_ (red line) and *h*_TOX_ (yellow line).

In a next step, we analyze the fabrication tolerance of our ideal grating coupler without metal mirror; see Figure 2. We sweep in 2D-FDTD the most important parameters, which are *h*_Si_, *h*_ILO_, Λ, FF, *h*_BOX_, and the thickness of the top oxide (*h*_TOX_). In each sweep, we kept the other parameters constant. In Figure 5a, the CE of the grating coupler is plotted in relation to *h*_Si_. Each color indicates a different *h*_ILO_. In (b), we plot the coupling efficiency as a function of *h*_ILO_. Each color indicates a different *h*_Si_. The individual simulations are indicated by colored dots. We find a 1-dB tolerance of 86 nm for *h*_Si_ and 139 nm for *h*_ILO_, indicating an exceptional large tolerance over the two parameters. In Figure 5c, the dependence of the CE on Λ (red line) and the FF (yellow line) are shown. The CE is reduced by 1 dB when varying Λ by ΔΛ = 34 nm and when varying the FF by as much as 0.1455, which corresponds to a detuning of *L*_M_ by as much as 129 nm. Finally, Figure 5d shows the CE in relation to *h*_BOX_ (red line) and *h*_TOX_ (yellow line). Varying *h*_BOX_ results in an oscillating behavior arising from constructive and destructive interference of the reflections at the interface of the buried oxide and the silicon handle wafer. From the yellow curve, it is seen that *h*_TOX_ behaves similarly, and a maximum CE is observed at 1.25 μm. Typically, there is a trade-off between efficiency and bandwidth.^29,30^ If higher optical bandwidth is required, one could potentially utilize an overlay grating material with a lower refractive index than silicon at the price of lower coupling efficiency.

### Dual Polarization O-Band Grating Coupler

In the next step, we design and demonstrate a dual-polarization (or polarization insensitive) 1D grating coupler for the O-band using the same principle as the O-band coupler described in the previous section. The use of O-band wavelengths has become the standard for intradatacenter interconnects due to the zero-dispersion window around 1310 nm.^31^ Furthermore, the dual-polarization capability of integrated components is essential for the realization of polarization multiplexing schemes or the compensation of polarization rotating effects in the system.

**Figure 6:**
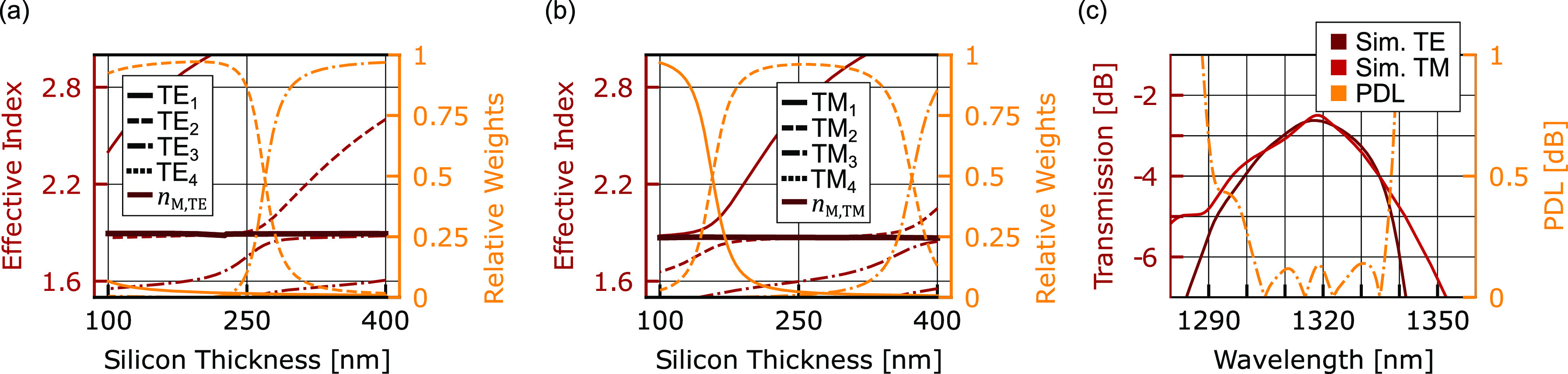
Polarization diverse 1D grating coupler enabled by the multilayer approach. (a) The *n*_eff_ (left) and the relative weights (right) of the modes TE_1_, TE_2_, TE_3_, and TE_4_ for the O-band in relation to *h*_Si_. (b) *n*_eff_ (left) and the relative weights (right) for the TM-polarized modes. (c) Optimized 1D grating coupler for TE and TM operation with a coupling efficiency above 2.5 dB with a polarization-dependent loss (PDL) of less than 0.25 dB for over 30 nm.

We overcome the polarization sensitivity of 1D grating couplers, which arises due to the birefringence of rectangular waveguides, by taking advantage of the large fabrication tolerances to exploit and the degree of freedoms provided by the overlay structure to find a low loss grating coupler for the TE as well as the TM modes. In contrast to the C-band grating coupler, there are not three but four modes in the multilayer structure because of the smaller wavelength. In Figure 6, the effective index (left) and the relative weights (right) of modes (a) TE_1_, TE_2_, TE_3_, and TE_4_ and (b) TM_1_, TM_2_, TM_3_, and TM_4_ is shown in relation to *h*_Si_. Similar to the C-band, the weighted average is almost constant. With the addition of TE_0_ and TM_0_, the effective indices in the grating region can be designed to be nearly equal for TE and TM polarization (*n*_GC,TE_ ≈ *n*_GC,TM_). This yields a similar pitch through the approximation of the phase matching equation. To achieve similar behavior in the C-band, control over *h*_BOX_ and *h*_SiN_ is necessary, which are fixed by the foundry in our work. We used the weighted average approximation described in the Concept section to calculate a starting point for the optimization routine. The result can be observed in Figure 6c. The simulated grating coupler operates for both polarizations with the same fiber angle and position, resulting in a CE of −2.6 dB. The polarization-dependent loss, defined as PDL = |CE_TE,dB_ – CE_TM,dB_|, is below 0.15 dB over a wavelength range of 30 nm. The grating has a pitch of Λ = 765 nm and a fill factor of 0.484. The thickness of the a-Si is 305 nm and of the interlayer oxide 100 nm.

### Fabrication

The a-Si overlay grating coupler was fabricated with a back-end-of-the-line process on a foundry-produced thick SiN platform. Amorphous silicon was deposited on a planarized sample with plasma-enhanced chemical vapor deposition, patterned with electron beam lithography, and etched by using inductively coupled reactive ion etching (ICP-RIE) in HBr plasma. The fabrication of the devices with metallic reflector included a backside oxide etch with ICP-RIE and a subsequent deep reactive ion etching (DRIE) of the Si substrate. Electron beam evaporation was used to deposit gold on the backside. Figure 1d shows a cross-sectional scanning electron microscopy (SEM) image generated with a focused ion beam of the grating coupler without metallic reflector. Figure 1b shows an optical microscopy image of the grating coupler with a metallic back reflector on the same chip. Finally, the chip was coated with poly(methyl methacrylate) (PMMA) as cladding material. It serves as an easy-to-deposit substitute for SiO_2_ with a comparable refractive index (*n* ≈ 1.48). We fabricated the dual-polarization grating coupler in the same way as the C-band gratings with *h*_Si_ = 305 nm and without a back reflector. The SEM image of the O-band grating coupler is found in Figure 1c.

## Results and Discussion

### Measurement of C-Band Grating Coupler

**Figure 7:**
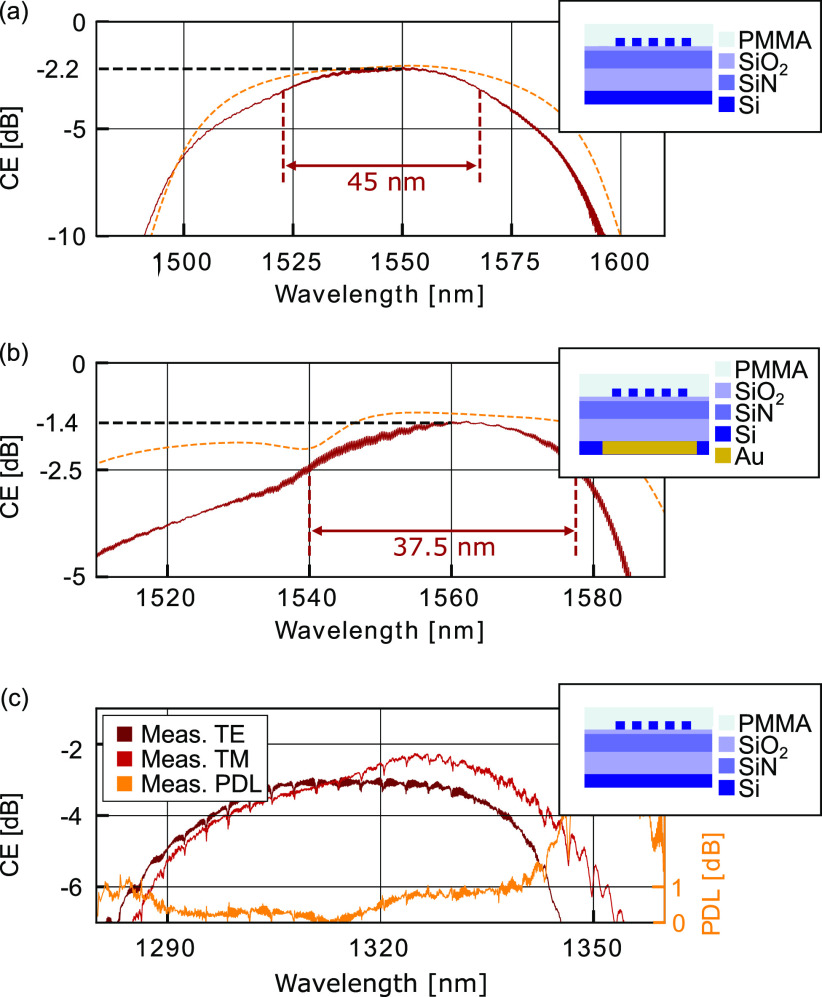
Measured (red line) and simulated (yellow line) coupling efficiency of the grating couplers versus simulated value with the 1 dB bandwidth. (a) CE of the TE C-band grating coupler without a back reflector. (b) CE of the TE C-band grating coupler with a back reflector. (c) Measurement result of the polarization diverse grating coupler with a measured PDL below 1 dB for over 40 nm.

Figure 7 shows the coupling efficiencies as a function of the spectra of the a-Si overlay grating couplers presented in this work. The red curves are from measurements. The yellow curves correspond to the simulated grating coupler. The parameters of (a) were adapted to the fabrication result through thickness measurements and SEM images of cross sections generated with a focused ion beam. For (b), we assume the same parameters with the only difference of a 50 nm thinner *h*_BOX_ due to over etching.

The plots in Figure 7a show the CEs of the a-Si overlay GCs without a back reflector. Coupling efficiencies of −2.2 dB were found with a 1 dB bandwidth of 45 nm. By adding a metallic back reflector, see Figure 7b, a CE of −1.4 dB and a 1 dB bandwidth of 37.5 nm was measured. The GCs of Figure 7a,b have identical geometries, and they stem from devices fabricated on the same chip. Characterization of the devices were conducted with optical transmission measurements using a tunable laser source in the C-band. A polarization controller was used to set the correct polarization. The setup losses were subtracted from the measurement and the SiN waveguides were assumed to be lossless. In practice we found propagation losses of ∼0.4 dB/cm. Yet, as our waveguides were as short as 1.5 mm, we disregarded such losses. Standard 90°-cleaved single-mode SMF28 fibers were used for coupling. The measurements and simulations of the a-Si GC are in very good agreement. The lower efficiency in the measured vs the simulated GC with metallic mirror potentially arises from utilizing the buried oxide as an etch stop for the DRIE process, which leads to a slightly thinner BOX thickness. Overetching had to be conducted to make sure all silicon is removed.

Table 1 summarizes different approaches for low loss grating couplers on the SiN platform and compares our measurements to the literature results. Our result of −2.2 dB is, to the best of our knowledge, the highest efficiency reported for a single-etch grating coupler without back reflector. The efficiencies of the a-Si grating coupler with a metallic mirror as shown in this work are comparable to the more complex DBR approaches.

**Table 1:** Overview of SiN Grating Coupler Results Found in the Literature

GC type	platform	mirror	CE (meas.; dB)	λ_max_ (nm)	CE (sim.; dB)	1 dB BW (nm)
multilayer PE DBR^14^	500 nm SiN	DBR	–1.17	1571	–0.45	40
multilayer FE DBR^14^	400 nm SiN	DBR	–1.24	1572	–0.31	39
multilayer Si-SiN^13^	400 nm SiN on SOI	Si gratings	–1.29	1536	–1.0	82
self-imaging stair-case GC^22^	600 nm SiN		–1.5	1550	–0.66	60 (3 dB)
multilayer FE DBR^16^	325 nm SiN	DBR	–1.75	1550	–1.0	76.34 (3 dB)
multilayer FE DBR^15^	400 nm SiN	DBR	–2.29	1573	–1.135	49
multilayer FE DBR^17^	400 nm SiN	DBR	–2.5	1490	–2.32	53
multilayer GC^19^	600 nm SiN on SOI	Si gratings	–2.5	1560	–2.13	65
multilayer GC^20^	220 nm dual-layer SiN		–2.56	1550	–2.28	54.1
FE GC^12^	400 nm SiN		–4.2	1570	–3.9	67
multilayer GC^32^	100 nm SiN		–5.0	1540	–3.8	75 (3 dB)
this work: a-Si overlay GC	800 nm SiN		–2.2	1550	–1.25	45
this work: a-Si overlay GC with mirror	800 nm SiN	metal	–1.4	1560	–0.42	37.5

### Measurement of O-Band Dual Polarization Grating Coupler

Figure 7c shows the measured results of −2.3 dB with a 32 nm 1 dB bandwidth for the TM polarization and −3.0 dB CE with a 41 nm 1 dB bandwidth for the TE polarization. The PDL is below 1 dB over a wavelength range of 40 nm. The measurement was conducted using a similar setup as for the C-band grating coupler with a tunable laser source in the O-band. Polarization-sensitive reference grating couplers were used to set and adjust the polarizations with a polarization controller. Analog to the C-band, we subtracted the setup losses and assumed that the SiN waveguides are lossless. The angle of the standard 90°-cleaved single-mode SMF28 fibers was set to 10.5° for both polarizations. The small dips in the spectrum originate from ring resonators coupled to the SiN waveguide. Table 2 summarizes the literature results for polarization diversity schemes with 1D grating couplers for reference. We find that our approach offers a significantly higher coupling efficiency than other 1D grating approaches on silicon or silicon nitride photonics. Furthermore, in contrast to literature, we do not see an efficiency penalty in comparison to single polarization grating couplers.

**Table 2:** Polarization Diversity Schemes for 1D GC on Si or SiN Found in the Literature

GC type	platform	CE (meas.; dB)	λ_max_ (nm)	PDL < 1 dB (meas.; nm)	PDL < 1 dB (sim.; nm)
multilayer PE GC^33^	polysilicon on SOI	–3.93	1550	77 (1.28 dB)	55
multilayer FE GC^18^	dual-layer 450 nm SiN on SOI	–4.8	1310	>100	69
zero-birefringence FE GC^34^	SOI	–6.1	1274	73	41
PE nonuniform GC^26^	SOI	–7.2	1570	22	70
this work: a-Si overlay GC	800 nm SiN	–2.3	1325	32	41

## Conclusion

In this work, we introduce highly efficient grating couplers for an emerging SiN platform. The single etch a-Si gratings have been fabricated by a back-end-of-the-line process at the wafer-scale on foundry-produced SiN waveguides. For TE-polarized light in the C-band, simulations yield high CEs of −1.11 without and −0.44 dB with metallic mirrors. In experiments, we found CEs of −2.2 and −1.4 dB, respectively. Furthermore, we demonstrate efficient and polarization-insensitive 1D grating couplers in the O-band. We measure a PDL below 1 dB within a 40 nm spectral window with a CE of −2.3 dB (TM) and −3.0 dB (TE) in the same device for coupling to a fiber aligned along the same fiber angle. Our approach offers a solution to a pending problem and provides ease of fabrication by taking advantage of a fabrication-tolerant design that is compatible with wafer-scale testing. The low fabrication complexity might help to further leverage the SiN platform.

## Abbreviations

SiN: silicon nitride
PICs: photonic integrated circuits
GC: grating couplers
BEOL: back-end-of-the-line
CE: coupling efficiency
EC: edge coupling
GC: grating coupling
PDL: polarization-dependent loss
ILO: interlayer-oxide
a-Si: amorphous silicon
FF: fill factor
TOX: top oxide
BOX: buried oxide
ICP-RIE: inductively coupled reactive ion etching
DRIE: deep reactive ion etching
SEM: scanning electron microscopy
PMMA: poly(methyl methacrylate)
DBR: distributed Bragg reflectors

## References

[ref1] Henry C. H.; Kazarinov R. F.; Lee H. J.; Orlowsky K. J.; Katz L. E. Low Loss Si_3_N_4_–SiO2 Optical Waveguides on Si. Appl. Opt. 1987, 26 (13), 2621–2624. 10.1364/AO.26.002621. 20489931

[ref2] Bucio T. D.; Lacava C.; Clementi M.; Faneca J.; Skandalos I.; Baldycheva A.; Galli M.; Debnath K.; Petropoulos P.; Gardes F. Silicon Nitride Photonics for the Near-Infrared. IEEE J. Select. Topics Quantum Electron. 2020, 26 (2), 1–13. 10.1109/JSTQE.2019.2934127.

[ref3] Heideman R.; Leinse A.; Hoving W.; Dekker R.; Geuzebroek D.; Klein E.; Stoffer R.; Roeloffzen C.; Zhuang L.; Meijerink A. Large-Scale Integrated Optics Using TriPleX Waveguide Technology: From UV to IR. Photonics Packaging, Integration, and Interconnects IX; SPIE, 2009; Vol. 7221, pp 203–217. 10.1117/12.808409.

[ref4] Wilmart Q.; El Dirani H.; Tyler N.; Fowler D.; Malhouitre S.; Garcia S.; Casale M.; Kerdiles S.; Hassan K.; Monat C.; Letartre X.; Kamel A.; Pu M.; Yvind K.; Oxenlowe L. K.; Rabaud W.; Sciancalepore C.; Szelag B.; Olivier S. A Versatile Silicon-Silicon Nitride Photonics Platform for Enhanced Functionalities and Applications. Applied Sciences 2019, 9 (2), 255 10.3390/app9020255.

[ref5] Puckett M. W.; Liu K.; Chauhan N.; Zhao Q.; Jin N.; Cheng H.; Wu J.; Behunin R. O.; Rakich P. T.; Nelson K. D.; Blumenthal D. J. 422 Million Intrinsic Quality Factor Planar Integrated All-Waveguide Resonator with Sub-MHz Linewidth. Nat. Commun. 2021, 12 (1), 934 10.1038/s41467-021-21205-4. 33568661 PMC7876138

[ref6] Pfeiffer M. H. P.; Herkommer C.; Liu J.; Morais T.; Zervas M.; Geiselmann M.; Kippenberg T. J. Photonic Damascene Process for Low-Loss, High-Confinement Silicon Nitride Waveguides. IEEE J. Sel. Top. Quantum Electron. 2018, 24 (4), 1–11. 10.1109/JSTQE.2018.2808258.

[ref7] Liu J.; Huang G.; Wang R. N.; He J.; Raja A. S.; Liu T.; Engelsen N. J.; Kippenberg T. J. High-Yield, Wafer-Scale Fabrication of Ultralow-Loss, Dispersion-Engineered Silicon Nitride Photonic Circuits. Nat. Commun. 2021, 12 (1), 2236 10.1038/s41467-021-21973-z. 33863901 PMC8052462

[ref8] Marchetti R.; Lacava C.; Carroll L.; Gradkowski K.; Minzioni P. Coupling Strategies for Silicon Photonics Integrated Chips [Invited]. Photon. Res., PRJ. 2019, 7 (2), 201–239. 10.1364/PRJ.7.000201.

[ref9] Fernández J.; Baños R.; Doménech D.; Domínguez C.; Muñoz P. Low-Loss Inverted Taper Edge Coupler in Silicon Nitride. IET Optoelectronics 2019, 13 (2), 62–66. 10.1049/iet-opt.2018.5065.

[ref10] Tummidi R. S.; Webster M. Multilayer Silicon Nitride-Based Coupler Integrated into a Silicon Photonics Platform with <1 DB Coupling Loss to a Standard SMF over O, S, C and L Optical Bands. 2020 Optical Fiber Communications Conference and Exhibition (OFC); OFC, 2020; pp 1–3.

[ref11] Zhu X.; Li G.; Wang X.; Li Y.; Davidson R.; Little B. E.; Chu S. T. Low-Loss Fiber-to-Chip Edge Coupler for Silicon Nitride Integrated Circuits. Opt. Express, OE 2023, 31 (6), 10525–10532. 10.1364/OE.483907. 37157597

[ref12] Doerr C. R.; Chen L.; Chen Y.-K.; Buhl L. L. Wide Bandwidth Silicon Nitride Grating Coupler. IEEE Photonics Technology Letters 2010, 22 (19), 1461–1463. 10.1109/LPT.2010.2062497.

[ref13] Sacher W. D.; Huang Y.; Ding L.; Taylor B. J. F.; Jayatilleka H.; Lo G.-Q.; Poon J. K. S. Wide Bandwidth and High Coupling Efficiency Si_3_N_4_-on-SOI Dual-Level Grating Coupler. Opt. Express 2014, 22 (9), 10938 10.1364/OE.22.010938. 24921792

[ref14] Nambiar S.; Ranganath P.; Kallega R.; Selvaraja S. K. High Efficiency DBR Assisted Grating Chirp Generators for Silicon Nitride Fiber-Chip Coupling. Sci. Rep 2019, 9 (1), 18821 10.1038/s41598-019-55140-8. 31827148 PMC6906413

[ref15] Nambiar S.; Kumar A.; Kallega R.; Ranganath P.; Selvaraja S. K. High-Efficiency Grating Coupler in 400 Nm and 500 Nm PECVD Silicon Nitride With Bottom Reflector. IEEE Photonics Journal 2019, 11 (5), 1–13. 10.1109/JPHOT.2019.2936430.

[ref16] Hong J.; Spring A. M.; Qiu F.; Yokoyama S. A High Efficiency Silicon Nitride Waveguide Grating Coupler with a Multilayer Bottom Reflector. Sci. Rep 2019, 9 (1), 12988 10.1038/s41598-019-49324-5. 31506482 PMC6736935

[ref17] Zhang H.; Li C.; Tu X.; Song J.; Zhou H.; Luo X.; Huang Y.; Yu M.; Lo G. Q. Efficient Silicon Nitride Grating Coupler with Distributed Bragg Reflectors. Opt. Express 2014, 22 (18), 21800 10.1364/OE.22.021800. 25321555

[ref18] Mak J. C. C.; Sacher W. D.; Ying H.; Luo X.; Lo P. G.-Q.; Poon J. K. S. Multi-Layer Silicon Nitride-on-Silicon Polarization-Independent Grating Couplers. Opt. Express 2018, 26 (23), 30623–30633. 10.1364/OE.26.030623. 30469956

[ref19] Xu P.; Zhang Y.; Shao Z.; Liu L.; Zhou L.; Yang C.; Chen Y.; Yu S. High-Efficiency Wideband SiN_x_-on-SOI Grating Coupler with Low Fabrication Complexity. Opt. Lett., OL 2017, 42 (17), 3391–3394. 10.1364/OL.42.003391. 28957045

[ref20] Ong E. W.; Fahrenkopf N. M.; Coolbaugh D. D. SiN _x_ Bilayer Grating Coupler for Photonic Systems. OSA Continuum 2018, 1 (1), 13 10.1364/OSAC.1.000013.

[ref21] Vitali V.; Lacava C.; Domínguez Bucio T.; Gardes F. Y.; Petropoulos P. Highly Efficient Dual-Level Grating Couplers for Silicon Nitride Photonics. Sci. Rep 2022, 12 (1), 15436 10.1038/s41598-022-19352-9. 36104372 PMC9474549

[ref22] Chen Y.; Bucio T. D.; Khokhar A. Z.; Banakar M.; Grabska K.; Gardes F. Y.; Halir R.; Molina-Fernández Í.; Cheben P.; He J.-J. Experimental Demonstration of an Apodized-Imaging Chip-Fiber Grating Coupler for Si_3_N_4_ Waveguides. Opt. Lett. 2017, 42 (18), 3566–3569. 10.1364/OL.42.003566. 28914903

[ref23] Fathololoumi S.; Hui D.; Jadhav S.; Chen J.; Nguyen K.; Sakib M. N.; Li Z.; Mahalingam H.; Amiralizadeh S.; Tang N. N.; Potluri H.; Montazeri M.; Frish H.; Defrees R. A.; Seibert C.; Krichevsky A.; Doylend J. K.; Heck J.; Venables R.; Dahal A.; Awujoola A.; Vardapetyan A.; Kaur G.; Cen M.; Kulkarni V.; Islam S. S.; Spreitzer R. L.; Garag S.; Alduino A. C.; Chiou R.; Kamyab L.; Gupta S.; Xie B.; Appleton R. S.; Hollingsworth S.; McCargar S.; Akulova Y.; Brown K. M.; Jones R.; Zhu D.; Liljeberg T.; Liao L. 1.6 Tbps Silicon Photonics Integrated Circuit and 800 Gbps Photonic Engine for Switch Co-Packaging Demonstration. Journal of Lightwave Technology 2021, 39 (4), 1155–1161. 10.1109/JLT.2020.3039218.

[ref24] Taillaert D.; Chong H.; Borel P. I.; Frandsen L. H.; De La Rue R. M.; Baets R. A Compact Two-Dimensional Grating Coupler Used as a Polarization Splitter. IEEE Photonics Technology Letters 2003, 15 (9), 1249–1251. 10.1109/LPT.2003.816671.

[ref25] Watanabe T.; Ayata M.; Koch U.; Fedoryshyn Y.; Leuthold J. Perpendicular Grating Coupler Based on a Blazed Antiback-Reflection Structure. J. Lightwave Technol., JLT 2017, 35 (21), 4663–4669. 10.1109/JLT.2017.2755673.

[ref26] Song J. H.; Doany F. E.; Medhin A. K.; Dupuis N.; Lee B. G.; Libsch F. R. Polarization-Independent Nonuniform Grating Couplers on Silicon-on-Insulator. Opt. Lett., OL 2015, 40 (17), 3941–3944. 10.1364/OL.40.003941. 26368681

[ref27] Kohli M.; Messner A.; Buriakova T.; Habegger P.; Chelladurai D.; Blatter T.; Smajic J.; Zervas M.; Fedoryshyn Y.; Koch U.; Leuthold J. Highly Efficient Grating Coupler for Silicon Nitride Photonics with Large Fabrication Tolerance. OSA Advanced Photonics Congress 2021; Optica Publishing Group, 2021; p IM4A.6. 10.1364/IPRSN.2021.IM4A.6.

[ref28] Kohli M.; Chelladurai D.; Buriakova T.; Moor D.; Eppenberger M.; Zervas M.; Fedoryshyn Y.; Koch U.; Leuthold J. Efficient Polarization-Insensitive O-Band Grating Couplers for Silicon Nitride. Optica Advanced Photonics Congress 2022 (2022); Optica Publishing Group, 2022; paper IM4B.1. 10.1364/IPRSN.2022.IM4B.1.

[ref29] Chen X.; Xu K.; Cheng Z.; Fung C. K. Y.; Tsang H. K. Wideband Subwavelength Gratings for Coupling between Silicon-on-Insulator Waveguides and Optical Fibers. Opt. Lett. 2012, 37 (17), 3483–3485. 10.1364/OL.37.003483. 22940923

[ref30] Passoni M.; Gerace D.; Carroll L.; Andreani L. C. Grating Couplers in Silicon-on-Insulator: The Role of Photonic Guided Resonances on Lineshape and Bandwidth. Appl. Phys. Lett. 2017, 110 (4), 041107 10.1063/1.4974992.

[ref31] Zhou X.; Urata R.; Liu H. Beyond 1 Tb/s Intra-Data Center Interconnect Technology: IM-DD OR Coherent?. Journal of Lightwave Technology 2020, 38 (2), 475–484. 10.1109/JLT.2019.2956779.

[ref32] Chmielak B.; Suckow S.; Parra J.; Duarte V. C.; Mengual T.; Piqueras M. A.; Giesecke A. L.; Lemme M. C.; Sanchis P. High-Efficiency Grating Coupler for an Ultralow-Loss Si_3_N_4_-Based Platform. Opt. Lett. 2022, 47 (10), 2498–2501. 10.1364/OL.455078. 35561384

[ref33] Zhou X.; Hu G.; Qin Y.; Tsang H. K. Polarization-Independent Waveguide Grating Coupler Using an Optimized Polysilicon Overlay. Opt. Lett. 2022, 47 (22), 5825–5828. 10.1364/OL.471717. 37219113

[ref34] Zhang B.; Schiller M.; Al Qubaisi K.; Onural D.; Khilo A.; Naughton M. J.; Popović M. A. Polarization-Insensitive 1D Grating Coupler Based on a Zero-Birefringence Subwavelength Corelet Waveguide. Opt. Lett. 2022, 47 (13), 3167–3170. 10.1364/OL.459306. 35776591

